# Toward unraveling reading–related modulations of tDCS–induced neuroplasticity in the human visual cortex

**DOI:** 10.3389/fpsyg.2014.00642

**Published:** 2014-06-20

**Authors:** Andrea Antal, Géza Gergely Ambrus, Leila Chaieb

**Affiliations:** ^1^Department of Clinical Neurophysiology, University Medical Center – University of GöttingenGöttingen, Germany; ^2^Department of Medical Psychology and Medical Sociology, University of GöttingenGöttingen, Germany; ^3^Institute of Psychology, Friedrich Schiller University of JenaJena, Germany; ^4^Clinic for EpileptologyBonn, Germany

**Keywords:** tDCS, visual cortex excitability, phosphene, TMS

## Abstract

Stimulation using weak electrical direct currents has shown to be capable of inducing polarity-dependent diminutions or elevations in motor and visual cortical excitability. The aim of the present study was to test if reading during transcranial direct current stimulation (tDCS) is able to modify stimulation-induced plasticity in the visual cortex. Phosphene thresholds (PTs) in 12 healthy subjects were recorded before and after 10 min of anodal, cathodal, and sham tDCS in combination with reading. Reading alone decreased PTs significantly, compared to the sham tDCS condition without reading. Interestingly, after both anodal and cathodal stimulation there was a tendency toward smaller PTs. Our results support the observation that tDCS-induced plasticity is highly dependent on the cognitive state of the subject during stimulation, not only in the case of motor cortex but also in the case of visual cortex stimulation.

## INTRODUCTION

Over the last 15 years transcranial direct current stimulation (tDCS) has become a promising tool in neuroplasticity research with perspectives in clinical neurophysiology (Nitsche and Paulus, 2011; Miniussi and Ruzzoli, 2013; Kuo et al., 2014). The primary effect of tDCS is a neuronal de- or hyperpolarization of transmembrane potentials (Creutzfeldt et al., 1962; Bindman et al., 1964), whereby the induced after-effects mainly depend on NMDA receptor-efficacy changes (Liebetanz et al., 2002). The most common way to evaluate cortical excitability changes induced by tDCS is by applying single-pulse transcranial magnetic stimulation (TMS) to the motor cortex (M1), since it allows the quantifiable measurements of its effects through the analysis of motor-evoked potentials (MEPs). In the resting muscle, anodal stimulation applied with 1 mA intensity for longer than 5 min increases the amplitude of MEPs, while cathodal stimulation decreases them (Nitsche and Paulus, 2000). With regard to the magnitude of the after effects induced by the stimulation besides the polarity, the combination of current strength – size of the stimulated area and duration of the stimulation are also relevant parameters (Agnew and McCreery, 1987; Batsikadze et al., 2013; Monte-Silva et al., 2013).

With regard to the visual cortex, it was shown in early animal experiments that the DC effect was less pronounced than on the M1, possibly due to the different cytoarchitecture, neurotransmitter level of the motor and visual areas and different spatial orientations of the neurons (Creutzfeldt et al., 1962). Later human studies confirmed these results, demonstrating that the tDCS after-effects are relatively short lasting in the visual areas compared to those of the M1, when using the same stimulation intensities and durations. Nevertheless, it was observed that the excitability of the visual cortex could be altered, as shown by the modulation of contrast thresholds (Antal et al., 2001; Kraft et al., 2010; Olma et al., 2011) and in the amplitude of the visual evoked potentials (Antal et al., 2004a; Accornero et al., 2007) after stimulation. The efficacy of tDCS over visual areas can also be demonstrated by measuring phosphene thresholds (PTs). TMS pulses delivered to the visual cortex can elicit visual sensations, called phosphenes (Meyer et al., 1991). The mean TMS intensity required to elicit phosphenes over multiple trials is defined as the PT. PT values are stable within subjects across time (Boroojerdi et al., 2000a, b). Therefore, the measurement of PTs is a frequently applied method in visual studies as a physiological index of cortical excitability, both in healthy participants and in clinical populations (Aurora et al., 1998, 2003; Mulleners et al., 2001; Brighina et al., 2002; Gerwig et al., 2005; Chadaide et al., 2007; Kammer and Baumann, 2010). In a previous work, we have elicited phosphenes by applying short trains of 5-Hz repetitive TMS delivered over the primary visual cortex (V1; Antal et al., 2003a). We found that cathodal stimulation over the V1 significantly increased PTs, probably due to diminished cortical excitability. Anodal stimulation resulted in the opposite effect, probably via induction of cortical hyperexcitability.

Previous studies suggested that a mental or physical activity in combination with tDCS applied over the M1 can modify the direction of stimulation-induced after-effects (Quartarone et al., 2004; Antal et al., 2007; Bradnam et al., 2010; Miyaguchi et al., 2013). Motor imagery undertaken following stimulation prolonged the effect of cathodal stimulation and abolished the effect of anodal stimulation (Quartarone et al., 2004). In another study, anodal stimulation combined with motor activity became inhibitory, while a cognitive task canceled the effect of both stimulation polarities (Antal et al., 2007). The effect of stimulation over M1 is highly dependent on the state (resting or active) of the muscle during stimulation (Bradnam et al., 2010; Miyaguchi et al., 2013). With regard to the combined stimulation of the visual cortex and any kind of activity, we are not aware of any published data. Therefore, the aim of this study was to investigate, whether a simple reading task can interact with tDCS applied over the visual cortex. We hypothesized that reading increases cortical excitability, and when combined with anodal stimulation this effect will be amplified. However, when it is in combination with cathodal stimulation, the net cortical excitability will not change.

## MATERIALS AND METHODS

### SUBJECTS

Twelve healthy volunteers (six male; aged between 18 and 30 years) were informed about all aspects of the study and gave written consent to participate. None of the subjects suffered from any neurological and psychological disorders, and neither had metallic implants/implanted electric devices nor took any medication regularly. The study conformed to the Declaration of Helsinki and the experimental protocol was approved by the local Ethics Committee.

### TRANSCRANIAL DIRECT CURRENT STIMULATION

Direct currents were transferred via a pair of saline-soaked surface sponge electrodes (5 cm × 7 cm) fixed to the scalp and delivered by a specially developed battery-driven current stimulator (NeuroConn GmbH, Ilmenau, Germany). One electrode was placed over the V1 (3 cm above the inion), while the other electrode was located at the Cz EEG position. The type of stimulation (anodal or cathodal) refers to the polarity of electrode above the V1. The subjects and investigators were blinded as to the polarity of tDCS in each experimental session. The current was applied for 10 min with an intensity of 1.0 mA. The fade in/fade out interval was set to 8 s. With regard to the sham stimulation there was only a 30 s stimulation with anodal or cathodal polarity.

### PT MEASUREMENTS

PTs were elicited using a MagPro-Stimulator (Medtronic Functional Diagnostics, Skovlunde, DK butterfly coil: MC-B70, biphasic pulse) and were measured before and after the tDCS sessions in order to determine the excitability of the V1. During the measurement, participants were seated in a comfortable chair. The coil was first positioned 2 cm over the inion with the handle pointing upward. In order to find the lowest PT (the best position for each individual participant) the coil was first moved to the left and then right side of the head in 1 cm steps. The optimal position was marked and measured using a measuring tape. Thereafter the stimulation was increased to suprathreshold intensity and was slowly decreased in 5%-steps until the participant reported the absence of the phosphenes. Around this value, the intensity of the stimulation was decreased and increased in 1%-steps until the participants reported seeing a visual sensation. This procedure was repeated four times and the intensity of the stimulation was recorded at each time. The means of PTs at a given time point were entered into the statistical analysis (see below).

### EXPERIMENTAL PROCEDURE

The experimental sessions were conducted in a repeated measurement design using a randomized order, with a break of at least 4 days between each session. There have been four experimental conditions: sham stimulation without reading, sham stimulation with reading, anodal stimulation with reading, and cathodal stimulation with reading. The volunteers were asked to bring a book that they are currently reading in their leisure time (this criteria excluded e-books, newspapers, and study material). The participants were seated in a comfortable chair. First the baseline PT measurement was performed. This was followed by the stimulation phase of the experiment, where, depending on the condition, subject received active stimulation combined with reading, or sham stimulation with or without reading (in the latter case, participants were instructed to sit passively during the stimulation phase, simply looking at the wall). PT measurements were repeated immediately, 10 and 20 min post-stimulation.

### STATISTICAL ANALYSIS

The PT values were normalized to the baseline. Repeated-measures ANOVAs were used to test for differences in PTs with the factors CONDITION (sham vs. sham + reading; anodal vs. sham + reading; cathodal vs. sham + reading) and TIME (0, 10, and 20 min post-stimulation). Conditional on significant *F*-values, Fisher LSD test was used to describe the main effects or interactions as revealed by the ANOVA. A *p-*value of = 0.05 was considered significant. Data are given as mean ± SEM.

## RESULTS

All of the subjects tolerated tDCS and no side effects were reported during or after the stimulation.

**FIGURE 1:**
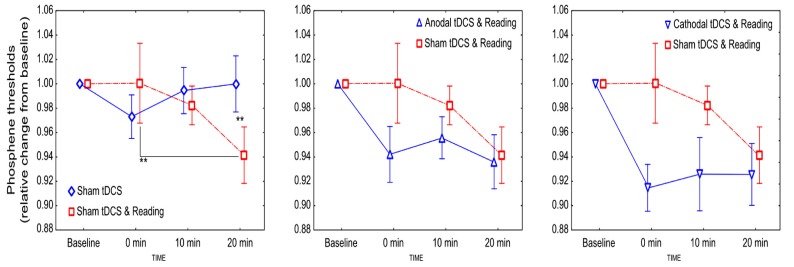
**FIGURE 1. The effect of tDCS combined with reading on the excitability of the visual cortex.** Values are normalized to baseline. Stars denote significant within- and between-condition differences. Error bars represent ±SEM.

With regard to the sham vs. sham + reading comparison rmANOVA revealed no main effect of CONDITION [*F*(1,11) = 0.25, *p* = 0.63, $\eta_{p}^{2}$ = 0.06]. A significant CONDITION and TIME interaction was found [*F*(2,22) = 4.31; *p* = 0.027, $\eta_{p}^{2}$ = 0.29]. The post hoc test revealed a significant PT decrease 20 min post-stimulation in the sham + reading condition compared to the sham condition (*p* = 0.009), and a reduction in PT values within the sham + reading condition between the measurements taken at 0 and 20 minutes post-stimulation (*p* = 0.009; **Figure 1**). 

When real stimulation was combined with reading, the PTs decreased independently from the stimulation polarity (**Figure 1**). Concerning the comparison of the sham + reading and anodal + reading conditions, rmANOVA revealed no significant effect of CONDITION [*F*(1,11) = 2.37, *p* = 0.15, $\eta_{p}^{2}$ = 0.18], TIME [*F*(2,22) = 2.55, *p* = 0.1, $\eta_{p}^{2}$ = 0.19] and CONDITION × TIME interaction [*F*(2,22) = 1.16; *p* = 0.33, $\eta_{p}^{2}$ = 0.09], although a small reduction in the PTs in the anodal + reading condition was observed.

Concerning the comparison of the sham + reading and cathodal + reading conditions, rmANOVA revealed a significant main effect of CONDITION [*F*(1,11) = 7.15, *p* = 0.022, $\eta_{p}^{2}$ = 0.39], showing that the PT values after cathodal stimulation were smaller compared to the sham + reading condition. However, the main effect of TIME [*F*(2,22) = 1.72, *p* = 0.21, $\eta_{p}^{2}$ = 0.14] and the CONDITION × TIME interaction [F(2,22) = 2.07; *p* = 0.15, $\eta_{p}^{2}$ = 0.16] were not significant.

## DISCUSSION

In the present study, we have demonstrated that reading alone can increase cortical excitability significantly. Furthermore, tDCS-induced neuronal plasticity over V1 was modified by reading: instead of showing the previously observed bidirectional response of anodal and cathodal stimulations on PTs (Antal et al., 2003a, b), application of both stimulation polarities resulted in a tendency toward an increase in cortical excitability that was significant after cathodal stimulation compared to sham. In a previous study examining task modulation of tDCS effects over the M1, it was reported that tDCS-induced alterations in cortical excitability could be modified by task engagement, for example by paying attention to a mental activity (IQ test) or by repeated contractions of the target muscle during stimulation (Antal et al., 2007). In this study, during the passive condition, anodal stimulation increased whilst cathodal stimulation decreased the amplitude of MEPs, as described by many previous studies. However, when performing a motor exercise, the M1 excitability was lower both after anodal and cathodal stimulation, when compared to the resting condition. Since the execution of a motor task alone can result in a decrease in cortical excitability, it was suggested that the stimulation here had no effect during the combined (tDCS + motor exercise) condition. We suppose that in the present study the significant *increase* in cortical excitability is due to the combined effect of the reading process and the electrical stimulation. Although phosphene perception can be used as a biomarker, when measuring visual cortical excitability (like the MEP amplitude with regard to the M1), PT detection is probably not a close enough analog to the MEP (Taylor et al., 2010). Furthermore, in the present study an increase in cortical excitability was observed after reading alone. Anodal stimulation probably facilitated this effect by further enhancing synaptic functioning. However, it is difficult to explain the excitability increasing effect of cathodal stimulation. This effect can be related to an increase in the signal-to-noise-ratio (Antal et al., 2004b) or in combination with reading, may induce metaplastic-like effects (Karabanov and Siebner, 2012).

Although the number of currently available studies assessing the effects of tDCS over the V1 is still limited, it has been repeatedly demonstrated that direct currents can alter visual performance bidirectionally (Antal et al., 2001, 2003a, b; Accornero et al., 2007; Kraft et al., 2010; Olma et al., 2011). However, the results are still contradictory. Efforts have been made to enhance our understanding of the reasons behind the reported heterogeneous effects, e.g., by explaining the impact of electrode position, target area of stimulation, and the type of task. Nevertheless, we suggest that one of the contributing factors behind the contradictory results might come from the fact that the experimental conditions during stimulation were not standardized (e.g., in several laboratories during an experimental session a variety of activities are permitted: for example, reading, surfing the internet, and eating are often allowed both during the stimulation itself and during pauses between repeated measurements, see also recent review, Horvath et al., 2014). Further studies systematically probing not only stimulation parameters but environmental effects might be needed to explore the reasons for the inconsistencies among studies examining electrical stimulation effects over the visual cortex.

## Conflict of Interest Statement

The authors declare that the research was conducted in the absence of any commercial or financial relationships that could be construed as a potential conflict of interest.
